# The Structure and Nephroprotective Activity of Oligo-Porphyran on Glycerol-Induced Acute Renal Failure in Rats

**DOI:** 10.3390/md15050135

**Published:** 2017-05-09

**Authors:** Jing Wang, Yun Hou, Delin Duan, Quanbin Zhang

**Affiliations:** 1 Key Laboratory of Experimental Marine Biology, Institute of Oceanology, Chinese Academy of Sciences, Qingdao 266071, Shandong, China; jingwang@qdio.ac.cn (J.W.); houyun.china@gmail.com (Y.H.); dlduan@qdio.ac.cn (D.D.); 2 Laboratory for Marine Biology and Biotechnology, Qingdao National Laboratory for Marine Science and Technology, Qingdao 266071, China; 3 Graduate University of the Chinese Academy of Sciences, Beijing 100049, China; 4 State Key Lab of Seaweed Bioactive Substances, Qingdao 266500, China

**Keywords:** sulfated galactan, oligo-porphyran, acute renal failure, gender difference

## Abstract

Porphyran is a sulfate galactan in the cell wall of *Porphyra*. Its acid hydrolysis product, oligo-porphyran (OP), was prepared and the structure studied by electrospray ionization time-of-flight mass spectrometry (ESI-TOF-MS). This oligosaccharide was mainly composed of monosulfate-oligo-galactan, disufate-oligo-galactan, trisulfate-oligo-galactan, trisulfate oligo-methyl-galactan, and 3,6-anhydrogalactose with the degree of polymerization ranging from 1 to 8. The effects of OP were investigated in the glycerol-induced acute renal failure (ARF) model. Compared with the normal group, rats from the glycerol-induced group exhibited collecting duct and medullary ascending limb dilation and casts. The OP-treated group exerted a protective effect against glycerol-induced changes. The results showed that the administration of OP markedly decreased mortality in female ARF rats. For male ARF rats, all of which survived, OP significantly decreased the blood urea nitrogen and serum creatinine levels. Ion levels in plasma and urine were significantly changed in ARF rats, whereas OP treatment almost recovered ion levels back to normal. This study showed a noticeable renal morphologic and functional protection by OP in glycerol-induced ARF rats.

## 1. Introduction

Acute renal failure (ARF) is a syndrome characterized by a rapid loss of kidney function [1]. During ARF, tubular fluid flow rate and glomerular filtration rate decrease rapidly. Subsequently, the kidney is unable to produce sufficient amounts of urine, and nitrogen-containing waste substances accumulate in the blood [2,3,4]. ARF is a contributor to many other diseases, including cardiovascular diseases, hypertension, and diabetes mellitus [5]. This syndrome has a major impact on the life quality of patients, and it is even life-threatening in some conditions [6].

Porphyran, with a linear backbone of alternating 3-linked β-d-galactose and 4-linked α-l-galactose-6-sulfate or 3,6-anhydro-α-l-galactose (3,6-AG) units [7], is the main polysaccharide in the cell wall of *Porphyra*. Porphyran has various biological activities, which are mainly attributed to its sulfated group [8,9]. In our previous study, we found that the sulfated polysaccharides from *Laminaria* and its hydrolysis products had protective effect on the rats with renal impairment [10,11]. One of an important factor for glycerol-induced renal injury is oxidative stress [12,13,14]. Porphyran has been proven to have strong antioxidant activity in vitro, however, few people studied its inhibition effect on ARF. Glycerol was a common agent for the induction of ARF in vivo and can lead to renal oxidative stress, as previously reported [15]. Therefore, the protective effect on glycerol-induced ARF of porphyan is obvious. 

Researchers found many factors of sulfated polysaccharides, such as molecular weight, degree of sulfation, and type of sugar, that influence their antioxidant activity. The earlier studies in our laboratory showed that different molecular weights of porphyan could influence the antioxidant activity [16]. Lower molecular weight porphyan exhibited stronger antioxidant activity compared with native porphyan. In this paper, we prepared oligo-porphyran by acid hydrolysis and evaluated the structure and effect of oligo-porphyran on the glycerol-induced ARF in rats. Dexamethasone was used in the positive control group due to its anti-inflammatory and antioxidant property in ARF rats [6]. The levels of blood urea nitrogen (BUN), serum creatinine (SCR), and ions in the rats’ blood and urine were investigated. The aim of this study was to clarify the structure and nephroprotective activity of oligo-porphyran on glycerol-induced acute renal failure in rats.

## 2. Results

### 2.1. The Structure Study of Oligo-Porphran

#### 2.1.1. Preparation, Fraction, and Analysis of Oligo-Porphyran (OP)

The chemical composition of the oligo-porphyran (OP) used in this study was shown in Table 1. It contains 71.39% galactose, 7.05% 3,6-anhydrogalactose, and 18.32% sulfate groups. Its average molecular weight was 1431 Da. This oligosaccharide was mainly composed of sulfated galactan with the degree of polymerization ranging from 1 to 8.

**Figure 1:**
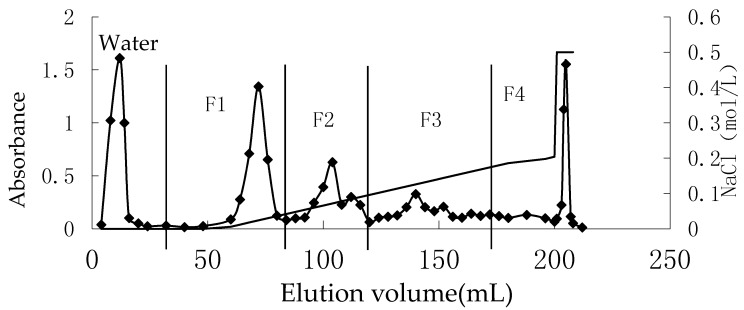
Chromatography of eluted OP on DEAE-sepharose CL-6B chromatography.

The eluting profile of chromatography of OP was shown in Figure 1. Three fractions were obtained: F1, F2, and F3 from 0 to 0.3 M NaCl elutions from column chromatography, and the F4 fraction was obtained from a 0.5 M NaCl elution. The sulfate group of F1–F4 was 16.54%, 16.06%, 18.98%, and 23.18%, respectively.

#### 2.1.2. Structure of Oligo-Porphyran (OP)

**Figure 2:**
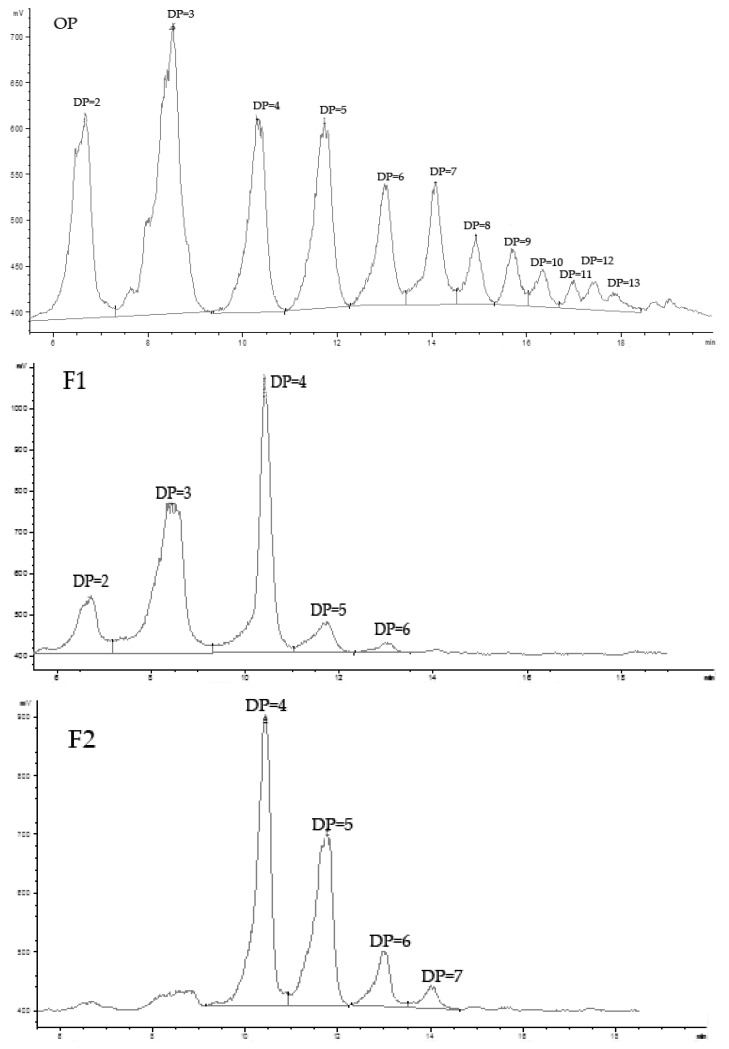

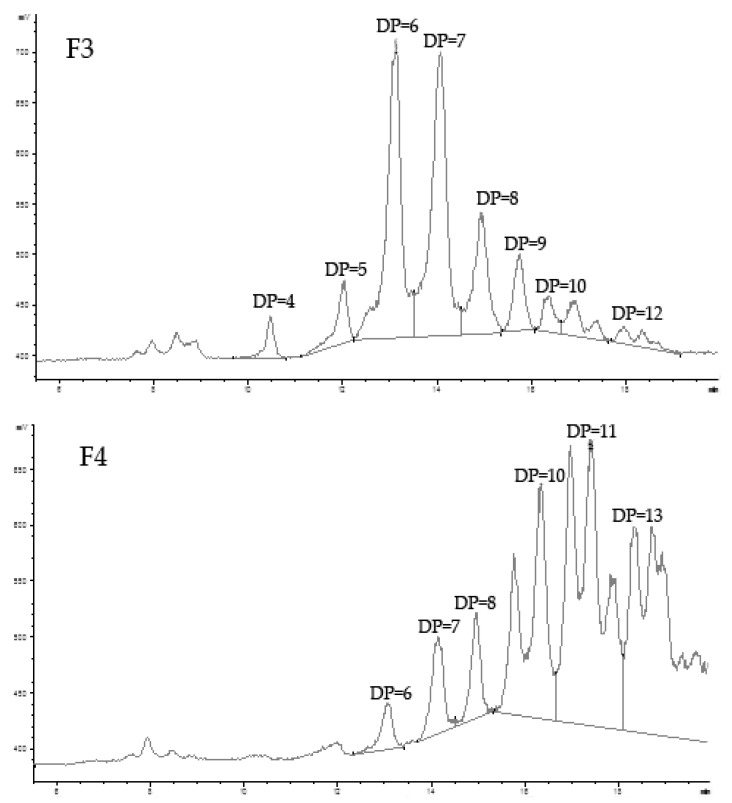
The degree of polymerization of OP and its fractions tested by HPLC.

The degree of polymerization (DP) of OP and its fractions F1–F4 was tested by HPLC (Figure 2). The number of sulfate groups and DPs of OP increased from F1 to F4. The sulfate group numbers of F1~F4 were 1, 2, 3, and >3, respectively, while the DPs of their main components were 2–4, 4–6, 6–8, and 7–13, respectively.

**Figure 3:**
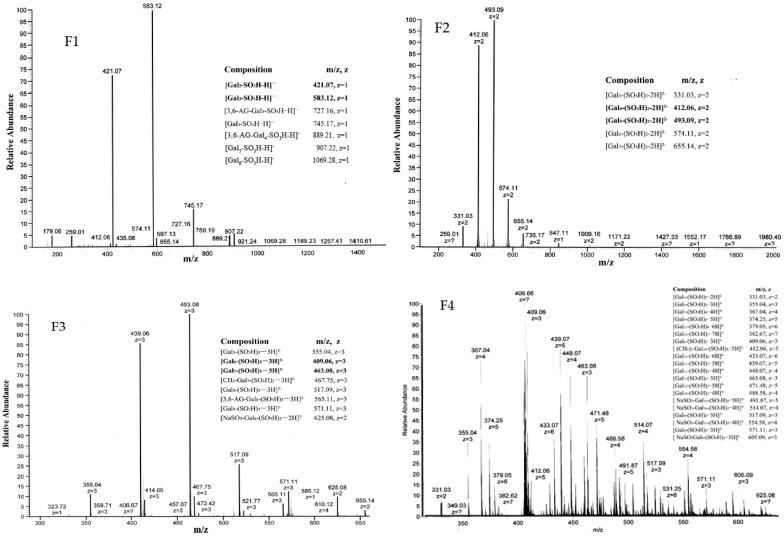
ESI-TOF-MS spectra of the fractions F1–F4 in a negative mode. All of the main peaks correspond to [M−*n*H]*^n^*^−^ of each individual sulfated galactan oligomer.

We studied the molecular weight distribution and structure of F1–F4 using ESI-TOF-MS (Figure 3). From the MS-spectrum of F1, we found a distribution of singly-charged ions corresponding to [Gal*_n_*-SO_3_H-H]^−^ (*n* = 2–6). There were less intensive signals, which were found to be [3,6-AG-Gal_*n*_-SO_3_H-H]^−^ (*n* = 3–4). Analysis of the spectrum of F2 found a distribution of doubly-charged ions corresponding to [Gal*_n_*-(SO_3_H)_2_-2H]^2−^ (*n* = 3–7), which means F2 was mainly made of disulfate-oligo-galactan. A distribution of trivalently-charged ions corresponding to [Gal*_n_*-(SO_3_H)_3_-3H]^3−^ (*n* = 5–9) were found in the MS spectrum of F3. Three ions at *m*/*z* 467.75 (−3), 565.11 (−3) and 625.08 (−3) were also detected in ESI-TOF-MS spectrum of F3, suggesting the presence of [CH_3_-Gal_7_-(SO_3_H)_3_-3H]^3−^, [3,6-AG-Gal_8_-(SO_3_H)_3_-3H]^3−^, and [NaSO_3_-Gal_6_-(SO_3_H)_2_-2H]^2−^. The molecular weight distribution and structure of F4 was complicated; the main ions at *m/z* 355.04 (−3), 367.04 (−4), 374.25 (−5), 409.06 (−6), 433.07 (−7), 439.07 (−5), 448.07 (−4), 463.08 (−3), 471.48 (−5), and 488.58 (−4) were identified as [Gal*_n_*(SO_3_H)*_m_*-mH]*^m^*^−^(*n* = 5–13, *m* = 3–7), while the less-intensive ions detected at *m*/*z* 331.03 (−2), 412.06 (−5), 491.87 (−5), 514.07 (−4), 517.09 (−3), 554.58 (−4), 517.11 (−3), and 605.09 (−3) corresponded to [Gal_3_(SO_3_H)_2_-2H]^2−^, [(CH_3_)_2_-Gal_10_-(SO_3_H)_5_-5H]^5−^, [NaSO_3_-Gal_12_-(SO_3_H)_5_-5H]^5−^, [NaSO_3_-Gal_10_-(SO_3_H)_4_-4H]^4−^, [Gal_8_-(SO_3_H)_3_-3H]^3−^, [NaSO_3_-Gal_11_-(SO_3_H)_4_-4H]^4−^, and [NaSO_3_-Gal_8_-(SO_3_H)_3_-3H]^3−^, respectively.

### 2.2. The Nephroprotective Activity of Oligo-Porphyran on Glycerol-Induced Acute Renal Failure in Rats

#### 2.2.1. The Mortality Effect of Oligo-Porphyran (OP) in ARF Rats

**Figure 4:**
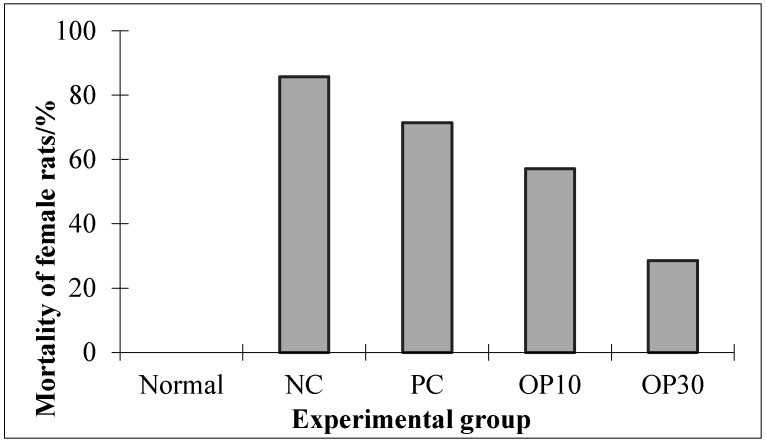
Mortalities of female rats in experimental groups. Normal: normal rats without acute renal failure (ARF); NC: negative control group (ARF rats); PC: positive control group (ARF + dexamethasone 0.1 mg/kg BW per day); OP10: ARF rats treated with oligo-porphyran (10 mg/kg body weight per day); OP30: ARF rats treated with oligo-porphyran (30 mg/kg body weight per day).

After the OP treated for seven days in ARF rats, all male rats in each group and female rats in the normal group survived, whereas deaths of female rats occurred in all ARF groups. The mortality of the negative control (NC) group was 85.71%. Dexamethasone reduced the mortality slightly to 71.43%. The ARF rats treated with 30 mg oligo-porphyran per body weight (BW) per day had the lowest mortality (28.57%), while the mortality of the OP10 group (ARF + oligo-porphyran 10 mg/kg BW per day) was 57.14% (Figure 4). 

#### 2.2.2. The BUN and SCR Level in ARF Rats

Before glycerol injection, we tested the BUN and SCR levels in all male and female rats, the average levels of BUN and SCR were 7.64 ± 0.71 nmol/L and 14.82 ± 0.84 μmol/L, respectively. Then the rats were randomly divided into five groups (seven male rats and seven female rats in each group). The rats were deprived of water for 24 h and received an intramuscular injection of 50% glycerol in NS into both hind limbs at a total dose of 10 mL/kg BW, except for the normal group, which was injected with NS. Then the animals were allowed access to a standard diet. Before the OP injection began at 48 h after glycerol injection, we tested the BUN and SCR levels to see whether the ARF models were successful (Table 2). From the data showed in Table 2, we could see the levers of BUN and SCR in ARF group were much higher than in the Normal group. Thus, we concluded the ARF models were successful. 

**Figure 5:**
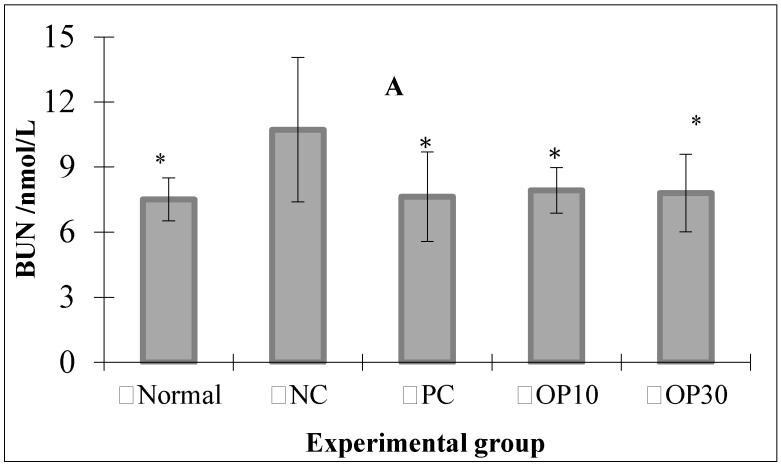

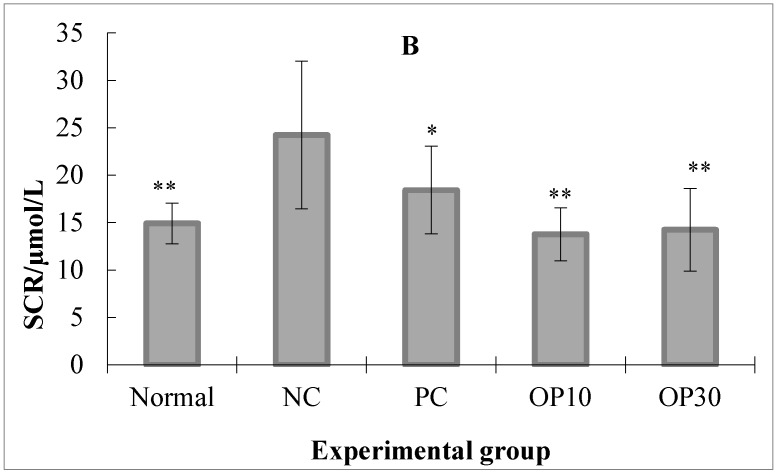
Blood urea nitrogen (BUN, Figure 5A) and serum creatinine (SCR, Figure 5B) levels of experimental groups. Normal: normal rats without acute renal failure (ARF); NC: negative control group (ARF rats); PC: positive control group (ARF + dexamethasone 0.1 mg/kg BW per day); OP10: ARF rats treated with oligo-porphyran (10 mg/kg body weight per day); OP30: ARF rats treated with oligo-porphyran (30 mg/kg body weight per day); * *p* < 0.05, ** *p* < 0.01 compared with NC. Data are presented as mean ± S.D. (*n* = 7).

As described in Figure 4, after the experiment, the mortality of the female ARF rats ranged from 28.57% to 85.71%, which means most of the female ARF rats died in NC, PC, and OP10 groups. From these results we suggest that the female ARF rats may not be suitable for studying the nephroprotective activity of OP with such high mortality. Thus, in the following study, we use just the data from male ARF rats to analyze the nephroprotective activity of the OP. Figure 5A shows that the levels of BUN in the normal, dexamethasone-treated ARF and two oligo-porphyran-treated ARF groups are significantly lower than that in the NC group (*p* < 0.05). The concentrations of SCR in the oligo-porphyran-treated groups are also significantly lower than that in the NC group in Figure 5B (*p* < 0.01). Comparing the rats in OP10 and OP30 groups, both OP10 and OP30 injections could lower the BUN and SCR levels (*p* < 0.01, respectively).

#### 2.2.3. The Ions and Protein Levels in the Urine and Plasma of ARF Rats

The ions and protein levels in the urine and plasma of each experimental group were analyzed (Table 3). In blood, OP treatment reduced only the concentration of Cl^−^ significantly (*p* < 0.05). The levels of albumin and other ions, such as Ca^2+^, Na^+^, and K^+^, did not change significantly by OP, although the normal, OP10, and OP30 groups had lower concentration of K^+^ than that of the NC group. In urine, it was noted that K^+^, Na^+^, and Cl^−^ levels in the normal, OP10, and OP30 groups were significantly higher than those in the NC group (*p* < 0.01).

#### 2.2.4. Histopathological Studies on Renal Tissues of ARF Rats

**Figure 6:**
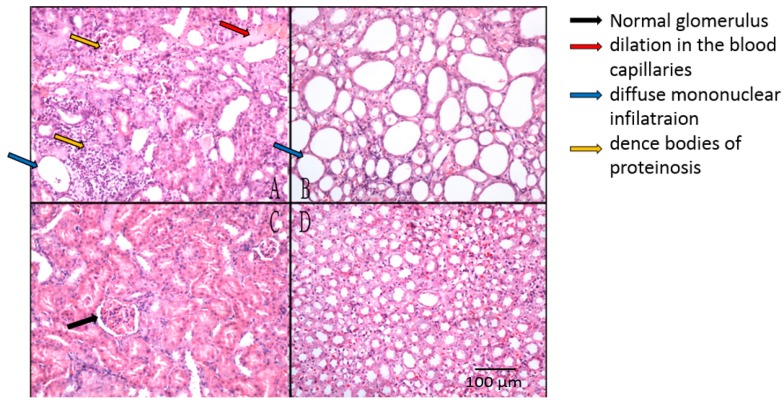
Histopathological studies on renal tissues of rats in the NC group (**A**,**B**) and OP10 (**C**,**D**) group. (**A**,**C**): cortex; (**B**,**D**): medulla. NC group: negative control group (acute renal failure rats); OP10 group: acute renal failure rats treated with oligo-porphyran (10 mg/kg body weight per day).

Renal tissues stained with hematoxylin and eosin were examined under light microscopy for the histopathological assay (Figure 6). The rats in the normal group were found to be in normal physiological condition. These animals had normal glomeruli with tubules (data not shown). However, the NC group rats had marked dilations in the blood capillaries of glomeruli and diffuse mononuclear infiltrations in the partial lymphocytes of glomeruli. Severe expansion and cast-shaped accumulations of dense bodies of proteinosis also appeared in the tubules (Figure 6A,B). With respect to the PC, OP10, and OP30 groups, no marked alteration was detected in the glomerulus, and normal corticomedullary demarcations were observed, although there were slight dilations in the tubules (Figure 6C,D). 

## 3. Discussion

We got an oligo-porphyran (OP) using acid hydrolysis method from porphyran, its average molecular weight was 1431 Da. The main composition of the OP were galactose (71.39%), 3,6-anhydrogalactose (7.05%), and sulfate group (18.32%), respectively. OP can be further classified using a DEAE Sepharose Fast Flow anion-exchange column and obtained four fractions (Figure 1). The degree of polymerization (DP), molecular weight distribution, and structure of F1–F4 were studied using HPLC and ESI-TOF-MS (Figure 2 and Figure 3). The DPs of F1–F4 was increased from two to 13, in accordance with the ESI-TOF-MS results. Analysis with the ESI-TOF-MS spectrums, we found F1 was mainly made of monosulfate-oligo-galactan, F2 was mainly made of disulfate-oligo-galactan, F3 was mainly made of trisulfate-oligo-galactan, trisulfate oligo-methyl-galactan, and trisulfate 3,6-anhydrogalactose. The molecular weight distribution and structure of F4 was complicated; it was mainly made of sulfate oligo-galactan, and the sulfate groups number from three to seven. The fractions F1–F4 were all mainly made of sulfate oligo-galactan, the differences were in the number of sulfate groups. From our previous knowledge and experiments, we know the sulfate group is an important factor which could influence the antioxidant activity of sulfated polysaccharides. However, this is not to say that higher is better. Thus, in this study we chose OP for the tested samples. We should confirm if OP had nephroprotective activity in vivo, firstly. If OP exhibited excellent nephroprotective activity, we will using fractions to study the relationship between the structure and the nephroprotective activity furthermore in the future. 

Glycerol-induced renal injury in rats, which closely resembles the lesions in the human kidney with ARF caused by transfusion accidents or crush injuries [2], is the most widely used model for studying acute renal failure. Therefore, glycerol injection was employed in the present study to develop ARF rat models. The results described above indicated that glycerol-induced rats lost their renal function efficiently. Compared with the normal group, the negative control group had significantly higher BUN and SCR levels (Figure 5), and its renal tissues were in much worse physiological condition.

As mentioned above, our previous study revealed that the sulfated polysaccharides from *Laminaria* and its hydrolysis products had an inhibitory effect on renal impairment [10,11]. We hypothesized that low molecular weight porphyran, a sulfated galactan, could also inhibit the development of renal failure in rats, and this speculation was verified by the present study. In general, the syndrome of ARF was characterized by a rapid reduction in the ability of the kidney to eliminate waste products, such as BUN and SCR. BUN is a nitrogen-containing waste that cannot be filtrated into the urine in renal failure diseases. SCR is the product of creatine high-energy phosphate consumption [17]. They are important indicators of ARF. The reduction of SCR and BUN underscores the improvement of renal filtration function. In our experiment, OP reduced the levels of BUN and SCR in ARF rats, the activity were similar, or better, than the PC group. This means OP treatment could be notably ameliorated by the impairment of renal filtration function. We also observed significant changes of electrolyte levels in serum and urine for the rats with ARF. However, the concentrations of K^+^, Na^+^, and Cl^−^ in the urine of the OP-treated groups were statistically higher than that in the NC group. According to this result, we can also conclude that the renal failure could be ameliorated by OP and the low concentration dose group exhibited excellent protection of renal function. Finally, we analyzed the renal histopathology of all of the rats, and the results demonstrated that OP reduced the lesion of kidneys compared with the NC group. Collectively, these results suggest that OP has a protective effect against glycerol-induced ARF. 

However, in this experiment, we only analyzed the renal function index at the sixth experimental day. From our results we obtained that the OP treatment has a positive effect on ARF rats, however, we still did not know on which day OP treatment had exhibited the renal protective effect because of a lack of data regarding the day-by-day observation. As for the findings in the histopathological study, we need to provide additional data from further study, which could indicate that the OP treatment cures the damaged tissues day by day. 

The fact that OP relieves glycerol-induced ARF might be due to the antioxidant activities of this sulfated saccharide. Many studies revealed that free radicals were critical mediators of heme-induced renal tubular damage and that antioxidants could be used to protect against renal failure [17,18]. Our previous report demonstrated that OP derived from *Porphyra* was an antioxidant, which had an inhibitory effect on superoxide, hydroxyl radicals, and hydrogen peroxide [16]. Therefore, the antioxidant activities of oligo-porphyran might contribute to its protective effect against ARF.

In this study, we also obtained an interesting result: the gender of rats affected their tolerance to the glycerol-induced ARF. Differing mortalities suggest that female rats were more sensitive to glycerol than male rats. The gender differences had been documented in the studies of other ARF models. For instance, female rats were more refractory to ischemic ARF than male rats [19], whereas female rats appeared more sensitive to cisplatin-induced ARF [20]. According to these studies, the sex hormone was considered an important reason for this difference. Our results indicated that OP reduced the mortality of female rats effectively (Figure 4). The mechanism of the differential mortality of the different sexes is still not clear, which will be the focus of future studies.

## 4. Materials and Methods

### 4.1. Preparation and Analysis of Oligo-Porphyran

Porphyran used for hydrolysis was extracted from *Porphyra yezoensis*, which was collected from Lianyungang (35°01′ N, 119°15′ E), China in 2015 [21]. For the preparation of oligo-porphyran, porphyran was dissolved in 0.5 mol/L H_2_SO_4_ and incubated for 2 h at 80 °C. After the acid hydrolysis, BaCl_2_ was added to neutralize the solution to pH 7.0. The solution was centrifuged, and then the supernatant was concentrated and lyophilized to obtain oligo-porphyran [22]. Oligo-porphyran was dissolved in normal saline (NS) for animal treatment.

The chemical characteristics of oligo-porphyran were investigated. Its total sugar content was analyzed with the phenol-sulphuric acid method [23]. The monosaccharide composition [24] and the molecular weight [22] of oligo-porphyran were determined by high-performance liquid chromatography. 3,6-anhydrogalactose content was analyzed according to the method of Yaphe [25]. Sulfate group content was determined using barium chloride-gelatin method [26]. The MWs of OP was determined by high-performance gel permeation chromatography with a TSK G3000PWxl column (TOSOH Co. Ltd., Tokyo, Japan). The mobile phase was 0.2 M Na_2_SO_4_ aqueous solution, and the flow rate was 0.5 mL min^−1^. The column temperature was maintained at 40 °C, and the samples were detected with a refractive index detector (Shimadzu RID-10A). We used dextran standards with molecular weights of 2.5 KDa, 7.8 KDa, 12.2 KDa, 21.4 KDa, 41.1 KDa, 84.4 KDa, 133.8 KDa, and 2000 KDa as standards. The dextran standards dissolved in 0.2 M Na_2_SO_4_ aqueous solution to form a 1% solution and added 20 µL each time. We used the molecular weight and the retention time to make the standard curve. The OP samples were analyzed using this standard curve. All data were recorded and processed using Shimadzu LC-Solution software [22].

### 4.2. Fraction of Oligo-Porphyran OP

0.5 g OP was dissolved in 2 mL water and then filtered through a microporous membrane (0.45 μm) before injecting into a DEAE Sepharose Fast Flow anion-exchange column (2.8 × 40 cm). The glass column (2.8 × 40 cm) was bought from Huideyi Company (Beijing, China) and DEAE Sepharose Fast Flow gel from GE Company (Boston, MA, USA), and then the column was filled with DEAE Sepharose Fast Flow by ourselves. Following loading of the sample, the column was pre-equilibrated with distilled water and then eluted by NaCl solution, gradiently, from 0 to 0.3 M at a flow rate of 1.5 mL min^−1^. Then eluted by 0.5 M NaCl solution. The elution was monitored by the concentration of total sugar by the phenol-sulfuric acid method at 490 nm [23], and pooled into four fractions (F1–F4) as illustrated in Figure 1. A Sephadex G10 column (GE Company, Boston, MA, USA) was then used to desalinize the four fractions.

### 4.3. The Structural Analysis of Oligo-Porphyran

The degree of polymerization of OP, and its fractions F1–F4, was tested by HPLC [27]. A Mal-CF column was bought from the Dalian Institute of Chemical Physics (CAS, DaLian, China), and a silica-maltose and evaporative light-scattering detector were used. Chromatographic conditions were as follows: oven temperature, 30 °C; sample concentration, 10 mg/mL; sample volume, 10 μL; mobile phase buffer, 100 mmol/L ammonium formate-formic acid (pH 3.1); flow rate, 1 mL/min; and the elution conditions were as shown in Table 4.

MW distributions of F1–F4 were determined by MS analysis with an LTQ Orbitrap XL mass spectrometer (Thermo Scientific, Sparta, NJ, USA).

### 4.4. Experimental Animals and Treatments

SD rats were reared in standard conditions of humidity (50 ± 5%), temperature (22 ± 1 °C), and lighting (12 h light/day). The animals were allowed access to a standard diet containing 20% protein and water ad libitum during the experiments. In this study, all drugs were injected into the abdominal cavity at a dose of 5 mL/kg body weight (BW) per day. These experiments were conducted in accordance with the National Guide for the Care and Use of Laboratory Animals of China. 

The rats were deprived of water for 24 h and received an intramuscular injection of 50% glycerol in NS into both hind limbs at a total dose of 10 mL/kg BW. Then the animals were allowed access to a standard diet. The following experiments began at 48 h after glycerol injection, when the ARF models were stable. 

The rats were randomly divided into five groups (seven male rats and seven female rats in each group): a normal group, an ARF group as a negative control (NC), a positive control group (PC, ARF + dexamethasone 0.1 mg/kg BW per day), an OP10 group (ARF + oligo-porphyran 10 mg/kg BW per day), and an OP30 group (ARF + oligo-porphyran 30 mg/kg BW per day). All of these groups were administered by intraperitoneal injection with the samples at the dosages described above for six days, while the rats in the NC and normal groups were treated with the same volume of saline. Urine specimens were collected on the fifth and sixth experimental days. After urine sampling, the rats were anaesthetized with chloral hydrate at the dose of 10 mg/kg and killed after blood samples were taken from their hearts. The blood was centrifuged at 2250× *g* for 20 min at 4 °C to obtain plasma for the biochemical analysis. The kidneys were taken and fixed in 10% formalin.

### 4.5. Biological Measurement

The urinary and plasma protein was measured by using a Pierce Coomassie Protein Assay Kit (Thermo Fisher Scientific, Petaluma, CA, USA) [28]. The principle is that, when mixed with a protein solution, the acidic coomassie-dye reagent changes color from brown to blue in proportion the amount of protein present in the sample. We used 20 μL urine and blood, each extracted from rats, to test levels of ions, SCR, and BUN with an automated biochemical analyzer (CX5, Beckman Instruments, Brea, CA, USA). The ion selective electrodes (Beckman, Brea, CA, USA) was used to measure ions, such as Na^+^, K^+^, Ca^2+^, and Cl^–^. We used the 10–300 mM NaCl, 10–100 mM KCl, and 1–10 mM CaCl_2_ as standard sfor Na^+^, K^+^, and Ca^2+^, respectively. We used 10–400 mM NaCl as the standard for Cl^−^. An ISE is a sensor that determines the concentration of ions in a solution by measuring the current flow through an ion-selective membrane. The renal histopathology was investigated using the previous method [5].

### 4.6. Statistical Analysis

The Student’s *t*-test was used to determine the level of significance of differences between the NC group and the other experimental groups. A significant difference was accepted with *p* < 0.05.

## 5. Conclusions

This study revealed the fractions F1–F4 were all mainly made of sulfate oligo-galactan with the degree of polymerization ranging from 1 to 8 and the sulfated groups numbering 1–3. We also found the protective effect of OP against glycerol-induced ARF. OP improved the renal functions of ARF rats markedly, as shown by renal histology and lower levels of SCR and BUN. A higher survival rate of male rats suggests a gender-related dependency of the sensitivity of rats to glycerol-induced ARF. This initial study of the OP-treated ARF did not clarify the mechanism of oligo-porphyran’s protective effect and the gender differences in this ARF model, which will be the subject of future work.

## Figures and Tables

**Table 1:** The chemical analysis of OP.

Sample	Mw (Kda)	Sulfate Group (%)	Galactose (%)	3,6-anhydrogalactose (%)
OP	1.43	16.90 ± 0.51	59.93 ± 1.44	6.45 ± 0.17

**Table 2:** Levels of BUN and SCR in the blood of experimental groups after injection 50% Glycerol for 48 h.

Gender	Index	Normal	NC	PC	OP10	OP30
Male	BUN (nmol/L)	7.73 ± 0.27	11.48 ± 1.23 **	12.27 ± 0.74 **	11.96 ± 1.08 **	12.32 ± 2.11 **
	SCR (μmol/L)	15.32 ± 0.74	26.72 ± 3.18 **	24.48 ± 1.64 **	27.11 ± 2.54 **	26.22 ± 1.71 **
Female	BUN (nmol/L)	6.91 ± 0.54 (*n* = 7)	13.44 ± 0.88 ** (*n* =4)	13.18 ± 0.68 ** (*n* =4)	12.64 ± 0.67 ** (*n* = 5)	14.97 ± 1.29 ** (*n* = 5)
	SCR (μmol/L)	13.94 ± 1.05 (*n* = 7)	25.64 ± 1.94 ** (*n* =4)	24.99 ± 0.96 ** (*n* =4)	26.70 ± 1.27 ** (*n* = 5)	26.02 ± 1.13 ** (*n* = 5)

Normal: normal rats without acute renal failure (ARF); NC: negative control group (ARF rats); PC: positive control group (ARF + dexamethasone 0.1 mg/kg BW per day); OP10: ARF rats treated with oligo-porphyran (10 mg/kg body weight per day); OP30: ARF rats treated with oligo-porphyran (30 mg/kg body weight per day); ** *p* < 0.01 compared with the Normal group. Statistical analysis was performed using ANOVA. Data are presented as mean ± S.D. (*n* = 7 in male rats, *n* = 4–7 in female rats).

**Table 3:** Levels of protein, potassium ions, sodium ions, chlorine ions, and calcium ions in the blood (**A**) and urine (**B**) of experimental groups.

**A**					
**Group**	**Albumin (g/L)**	**K^+^ (mmol/L)**	**Na^+^ (mmol/L)**	**Cl^−^ (mmol/L)**	**Ca^2+^ (mmol/L)**
Normal	32.95 ± 2.27	5.48 ± 0.63	148.27 ± 0.81 **	106.43 ± 2.00 **	2.52 ± 0.13
NC	33.19 ± 1.34	6.27 ± 2.22	144.53 ± 2.04	99.41 ± 3.84	2.54 ± 0.23
PC	36.96 ± 1.79	7.03 ± 3.04	145.68 ± 1.98	97.04 ± 3.66	2.66 ± 0.19
OP10	32.84 ± 1.55	5.46 ± 0.49	144.24 ± 1.66	103.70 ± 1.99 *	2.49 ± 0.10
OP30	32.82 ± 1.53	5.12 ± 0.24	143.63 ± 1.35	103.55 ± 0.54 *	2.56 ± 0.07
**B**					
**Group**	**Albumin (g/L)**	**K^+^ (mmol/L)**	**Na^+^ (mmol/L)**	**Cl^−^ (mmol/L)**	**Ca^2+^ (mmol/L)**
Normal	0.41 ± 0.19	64.56 ± 10.27 *	228.48 ± 27.75 **	338.58 ± 68.46 **	3.67 ± 2.06
NC	0.29 ± 0.22	47.13 ± 10.60	74.07 ± 33.67	113.93 ± 54.31	5.00 ± 2.58
PC	0.62 ± 0.57	42.88 ± 17.50	43.33 ± 31.89 *	71.49 ± 66.48	2.16 ± 2.10 *
OP10	0.25 ± 0.12	62.19 ± 5.22 **	185.21 ± 29.06 **	278.41 ± 60.09 **	3.78 ± 2.40
OP30	0.29 ± 0.12	67.45 ± 4.19 **	201.38 ± 61.62 **	282.80 ± 054 **	4.09 ± 1.01

Normal: normal rats without acute renal failure (ARF); NC: negative control group (ARF rats); PC: positive control group (ARF + dexamethasone 0.1 mg/kg BW per day); OP10: ARF rats treated with oligo-porphyran (10 mg/kg body weight per day); OP30: ARF rats treated with oligo-porphyran (30 mg/kg body weight per day); * *p* < 0.05, ** *p* < 0.01 compared with NC. Statistical analysis was performed using ANOVA. Data are presented as mean ± S.D. (*n* = 7).

**Table 4:** Mobile phase gradient.

Time (min)	Acetonitrile (%)	Buffer (%)
0	80	20
30	40	60
40	10	90
